# Genome-Wide Identification and Comprehensive Analysis of the *PPO* Gene Family in *Glycine max* and *Glycine soja*

**DOI:** 10.3390/genes16010017

**Published:** 2024-12-26

**Authors:** Ziye Song, Bo Wang, Jia Liu, Nianxi Liu, Zhigang Yi, Zhi Li, Zhimin Dong, Chunbao Zhang, Yingshan Dong, Yuqiu Li

**Affiliations:** 1 College of Agronomy, Jilin Agricultural University, Changchun 130118, China; skysziye@163.com; 2 Soybean Research Institute, Jilin Academy Agricultural of Science, Changchun 130033, China; bowang1122@126.com (B.W.); lj_hope0459@aliyun.com (J.L.); lnx69@126.com (N.L.); yizhigang0504@126.com (Z.Y.); lizhi527@126.com (Z.L.); dongzhimin2005@126.com (Z.D.); cbzhang@cjaas.com (C.Z.)

**Keywords:** *Glycine max*, *Glycine soja*, *PPO*, gene family

## Abstract

Background: Polyphenol oxidases (*PPOs*) form a multigene family that is widely distributed in plants, animals, and insects. To date, *PPOs* have been identified in plants such as *Populus* L. and *Solanum tuberosum* L., but studies on *PPOs* in soybean (*Glycine max* (L.) Merr.) and wild soybean (*Glycine soja* Sieb. and Zucc.) remain limited. Methods: To clarify the nature, structure, evolution, expression pattern, and interaction network of *PPOs* in these plants, we performed bioinformatics analysis and evaluated the expression patterns of *PPOs* in soybean and wild soybean throughout the growth period and under salt stress. Results: We identified 17 and 15 genes belonging to the PPO family. These genes were distributed across chromosomes 7 and 6 and could be divided into three groups. Most of these genes only contained one coding sequence (CDS), and their gene structure, conserved motifs, and 3D structures were very similar. Although there were a few intraspecies gene duplications, 75 gene replication pairs between soybean and wild soybean were detected. A Ka/Ks analysis showed that the *PPOs* in these plants were mainly subjected to purity selection. Moreover, the expression of the *PPO* genes varied greatly during different stages of the growth period and under salt stress, showing high temporal and spatial specificity. The protein interaction networks of these genes appeared to be quite distinct. Through the interaction analysis of the candidate gene *GmPPO2* selected under salt stress, *Glyma.07G059000*, *Glyma.10G279000*, and *Glyma.03G167900* were identified as the candidate genes regulating salt stress tolerance in soybean. Conclusions: These findings provide a foundation for further research on the evolution of soybean and wild soybean, as well as the functions of the *PPO* gene family.

## 1. Introduction

Polyphenol oxidases (*PPOs*) are copper-containing enzymes that are widely distributed in nature. In plants, *PPOs* are well-known for their role in the browning reactions that occur after tissue damage [1]. In addition, *PPOs* play a critical role in plant photosynthetic processes and help plants resist biotic and abiotic stresses [2,3]. At a subcellular level, the majority of PPO enzymes are located on chloroplasts, which indicates that they are involved in photosynthetic reactions [4]. For instance, Chen et al. [5] constructed a VIGS silencing vector to suppress *PPO* expression in *Clematis*. The results revealed a significant increase in the content of chlorophyll a and chlorophyll b in plants expressing this vector, as well as a notable improvement in the photosynthetic rate when compared to the control. This indicated that *PPO* silencing can increase photosystem II (PSII) activity in plants. Concurrently, the expression of ATP synthase and ferredoxin-NADP^+^ reductase was found to be markedly elevated in *PPO*-silenced *Clematis* plants. These findings suggest that silencing *PPO* can enhance the light response in plants, thereby increasing the supply of ATP and NADPH in plants subjected to stress.

Additionally, numerous reports have documented that PPO enzymes confer resistance to both abiotic and biotic stresses [6,7]. For example, Soha et al. [8] observed that silencing the *PPO* gene in *Juglans regia* L. via RNA interference leads to the formation of necrotic spots, which in turn reduces the stress tolerance of these plants. In contrast, Mahanila et al. [9] demonstrated that overexpressing the *jrPPO* gene in *Solanum lycopersicum* L. enhances the plant’s resistance to invasion by the diamondback moth. It has been postulated that PPO enhances the resistance of plants to biological stress. Under the conditions of a pathogen attack, the expression of *PPOs* is induced and upregulated, leading to the production of quinones and other reactive substances. These substances react with each other to produce melanin, which prevents the spread of the pathogen and thus enhances the plant’s resistance to infection [10].

*PPOs* belong to a polygenic family, and the number and function of the genes in this family vary across different species [11]. For example, nine *PPO* genes have been identified in *Solanum tuberosum* [12]. Meanwhile, *Solanum lycopersicum* L. contains 7 *PPO* genes [13], while *Salvia miltiorrhiza* Bge.—as the species with the highest known number of *PPO* genes—can contain up to 19 [14]. Interestingly, *Selaginella moellendorffii* Hieron., a plant with one of the smallest known genomes, also contains 11 identified *PPO* genes [15]. While many species, like those mentioned above, have large *PPO* gene families, other plants exhibit a very limited number of *PPO* genes. For example, only one *PPO* gene has been identified in *Malus pumila* Mill., *Vitis vinifera* L., *Manihot esculenta*, and *Ricinus communis* L., and surprisingly, no *PPO* gene has been identified in *Arabidopsis thaliana* (L.) Heynh., a model plant species [15,16]. *Arabidopsis* is known for its high gene homology with other complex plants, which makes it a key model organism in plant research. However, the absence of *PPO* genes in *Arabidopsis* makes it challenging to study the function of *PPOs* in other species [17]. These variations in *PPO* gene presence or absence can be related to gene amplification or loss during the evolution of plant species.

In cultivated soybean, which was domesticated from wild soybean [17,18], the identification of *GmPPOs* (soybean *PPOs*) has been completed. However, a comprehensive analysis of these genes has not been thoroughly conducted. Moreover, *GsPPOs* (wild soybean *PPOs*) have not been identified. In this study, we re-identified the *GmPPO* genes and also identified *GsPPO* genes. Using bioinformatics methods, we compared the *PPOs* from soybean and wild soybean and preliminarily analyzed their expression and interaction networks to provide a reference for further studies on the function of the *PPO* gene family in soybean and wild soybean.

## 2. Materials and Methods

### 2.1. Identification of Gene Family Members

The genomic data of soybean (*Glycine max Wm82.a2. v1*) and wild soybean (*Glycine soja v1.1*) were obtained from Phytozome 13 (https://phytozome.jgi.doe.gov/pz/portal.html, accessed on 10 September 2024. Previous studies have shown that genes belonging to the *PPO* family contain three conserved structural domains: Tyrosinase (PF00264), PPO1_DWL (PF12142), and PPO1_KFDV (PF12143) [19]. We downloaded the HMM model from the InterPro website (https://www.ebi.ac.uk/interpro/search/sequence/, accessed on 10 September 2024), used the model to perform an HMM search, and obtained the search results. The intersection was considered, and the CDD database (https://www.ncbi.nlm.nih.gov/cdd/, accessed on 11 September 2024), SMART database (http://smart.embl.de/, accessed on 11 September 2024), and Pfam database (http://pfam.xfam.org/, accessed on 11 September 2024) were then used to further identify the conserved domains of the initially screened candidate protein sequences. The protein sequences of PPOs were obtained by eliminating protein sequences without conserved domains and distantly related protein sequences. To gain a better understanding, these genes were sorted according to their order on the chromosome and named in sequence.

### 2.2. Physicochemical Characteristics and Subcellular Localization

The isoelectric point and relative molecular mass of the PPOs were tested with Bioperl, and their amino acid length, isoelectric point, and molecular mass were obtained. The PPO sequences were added to WoLF PSORT (https://www.genscript.com/wolf-psort.html, accessed on 14 September 2024), and their subcellular localization was predicted.

### 2.3. Analysis of Conserved Domains and Gene Structure

The conserved domains of the high-quality soybean *PPO* gene family sequences were analyzed using the MEME 5.1.0 online tool (https://meme-suite.org/meme/, accessed on 18 September 2024); the number of conserved domains was set to 5 [20]. We used Pfam to search for motifs and obtained motif comments (Table S1). Untranslated region (UTR) and coding sequence (CDS) information was extracted from GFF3 annotation files, and the conserved domains and gene structures were visualized together with the TBtools software [21]. The bootstrap value was set to 1000, and the other parameters were set to the default values.

### 2.4. Multiple Sequence Alignment and Phylogeny

The ClustalW program in MEGA 7 was used to perform multiple sequence alignment analysis on the selected soybean and wild soybean PPO protein sequences, and the neighbor-joining (NJ) method was employed to construct a phylogenetic tree. The evolutionary relationship among the different PPOs was determined. The bootstrap value was set to 1000, and the other parameters were set to the default values. Bootstrap values were significantly correlated with the credibility of the analysis. Finally, the online tool Evolview 2.0 (https://evolgenius.info//evolview-v2/#login, accessed on 22 September 2024) was used to beautify and partition the phylogenetic tree. The ‘basic’ and ‘annotation’ functions of this tool were used to make the evolutionary tree more circular and change the color of the gene names and groups.

### 2.5. Chromosome Localization and Gene Duplication Analysis

To obtain the chromosomal location information of *PPO* gene family members, we used the relevant gene annotation file and MapChart 2.32 to create a chromosomal map for the *PPO* genes. Adobe Illustrator CS5 was then used to enhance the map, adjusting the layout according to chromosome length and gene start positions. Using a Perl script (File S1), we identified the tandem repeat gene pairs within the gene family. An intra-genome collinearity analysis was performed with McScanX (08-05-2012) using its default parameters, which allowed us to identify tandem repeats and large-segment gene duplications within the family (e-value < 1 × 10^−10^). The results of this analysis were visualized using Circos v0.69 for a more polished representation of the collinearity [22]. Additionally, the evolutionary homology of the *PPO* genes was analyzed through comparative genomics using McScanX in Python (Table S2). The ratio of the non-synonymous substitution rate (Ka) to the synonymous substitution rate (Ks) was calculated using TBtools v2.142 [21]. A Ka/Ks ratio > 1 indicates positive selection, a ratio = 1 suggests neutral selection, and a ratio < 1 indicates negative or purifying selection. The divergence time (million years ago, Mya) for the fragment repeat genes was calculated using the formula (Ks/2λ) × 10^−6^, where λ is 6.1 × 10^−9^ [23].

### 2.6. Analysis of Cis-Acting Elements in the Promoter Region

We obtained a 1500 bp sequence upstream of each *PPO* gene family member using Phytozome 13 (*Glycine max Wm82.a2. v1*) and then added the sequence to the Plant CARE online tool (http://bioinformatics.psb.ugent.be/webtools/plantcare/html/, accessed on 25 September 2024) [24] to extract information on the cis-acting elements present in the sequence (Table S3). This information was visualized through the GSDS 2.0 online website (http://gsds.gao-lab.org/, accessed on 25 September 2024) [25].

### 2.7. Gene Expression Pattern Analysis

To study the expression of *PPOs* in different tissues and organs, we extracted the soybean expression data ‘genes.fpkm_tracking’ from Phytozome 13 (fragment per kilobase of exon model per million mapped reads, FPKM) (Table S4) [26]. TBtools was employed to draw a heat map of the expression, and the built-in editor was used to enhance the heat map [21]. The parameters of the redrawn heat map were the same as those of the original heat map.

### 2.8. Plant Materials, Stress Treatments, and Tissue Sampling

The seeds of soybean (Williams 82) and wild soybean (ZYD000066) were provided by the Jilin Academy of Agricultural Sciences (Changchun City, Jilin Province, China). Full and smooth seeds were selected, sterilized with 75% ethanol, and then placed in a 25 °C incubator for germination. After germination, the seeds were cultivated in soil, and different tissues of the plant were sampled throughout the growth period after the seedlings had grown their first three compound leaves. Another group of seedlings was planted in sandy soil. After the third trifoliate leaf expanded, the control group was irrigated with 1 × Hoagland nutrient solution (Huogelan, China), whereas the treatment group was irrigated with 1 × Hoagland nutrient solution with NaCl. The molar mass ratio of NaCl to Na_2_SO_4_ was 1:1, and the concentration of Na^+^ was 45 mmol·L^−1^. Samples were collected at 3 h, 6 h, 12 h, 24 h, 48 h, and 72 h after irrigation and stored at −80 °C [27].

### 2.9. RNA Isolation and Quantitative Real-Time PCR Analysis

The Plant RNA Extraction Kit (Trans, Xiamen, China) was used to extract RNA from the plant samples, and the purity and concentration of the total RNA were determined using the Nanodrop system (Thermo Fisher Scientific, Bedford, MA, USA). According to the kit instructions, cDNA was synthesized using the Prime Script™RT Reagent Kit (Takara, Beijing, China). Real-time fluorescence quantitative PCR (qRT-PCR) was performed on each cDNA template using the TB Green Mix (Takara, Beijing, China). The PCR amplification conditions were as follows: 95 °C for 5 min, followed by 45 cycles of 95 °C for 10 s and 60 °C for 30 s in a 10 μL reaction mixture. Three replicates were prepared per sample, and the QuantStudio 6 Flex system (Thermo Fisher Scientific, Bedford, MA, USA) was used to carry out the reactions. Relative gene expression was calculated using the 2^−ΔΔCt^ method, with Tubllin serving as the internal reference gene (Table S5).

### 2.10. Protein 3D Structure Interaction Network Prediction

SWISS-MODEL (https://swissmodel.expasy.org/, accessed on 13 October 2024) was employed to predict the 3D protein structure of all the PPOs (use AlphaFold 2). STRING 12.0 (https://cn.string-db.org/, accessed on 13 October 2024) was used to predict the protein interactions of the GmPPOs (not more than 10 interactors), and the software also provided annotations for the functions of the predicted proteins. The homologous gene mapping data of soybean and wild soybean predicted by OrthoVenn3 2022 (https://orthovenn2.bioinfotoolkits.net, accessed on 15 October 2024) were integrated, and the protein interaction network was obtained [28]. The Cytoscape 3.8.2 software was employed to enhance the protein interaction network. AlphaFold 3 was used to validate some of the STRING results [29]. Among them, Chain pair pae min: [num, chains, num, chains] array. The element (i, j) is the minimum PAE value between chain i and chain j, which is related to whether the two chains interact. Chain ptm: evaluate the PTM score of a single chain. Score: select the maximum value from chain pair pae min as the interaction score [29]. Protein function annotation was obtained from STRING.

## 3. Results

### 3.1. Identification and Distribution

The genomic data of the soybean and wild soybean obtained from the Phytozome database were compared with the Pfam search results for the three conserved domains, and 19 and 16 *PPO* candidate genes were initially screened. Then, candidate genes were validated using the CDD, SMART, and Pfam databases. Sequences lacking complete domains or with distant relationships were excluded. Finally, 17 genes from the soybean and 15 genes from the wild soybean, each containing three conserved domains, were selected. These genes were named *GmPPO1* to *GmPPO17* (Table 1) and *GsPPO1* to *GsPPO15* (Table 2) based on their chromosomal positions.

The analysis of protein physicochemical properties revealed that except for GmPPO1 (which only had 274 amino acids), the lengths of the other soybean PPO proteins ranged from 521 to 620 amino acids. Meanwhile, in the wild soybean, the protein lengths varied from 349 to 638 amino acids (Table 1 and Table 2). A similar trend was observed for the relative molecular masses of the proteins. Although the molecular mass of GmPPO1 was smaller, the relative molecular masses of the other proteins were relatively similar. The theoretical isoelectric points (pI) of the soybean proteins ranged from 5.84 to 8.68, with GmPPO1 having a pI of 5.84 and GmPPO14 having a pI of 8.68. The remaining proteins had a pI ranging from 6 to 8. In the wild soybean, the theoretical isoelectric point of PPO proteins was approximately 5.6 to 8.16, with GsPPO14 having a pI of 5.6 and GmPPO14 having a pI of 8.16. Meanwhile, the other wild soybean proteins also had pI values ranging between 5 and 8, indicating a shift from acidic to alkaline properties across the family.

The prediction of subcellular localization indicated that GmPPO1 was primarily located in the cytoplasm, GmPPO7 in the nucleus, and GmPPO16 and GmPPO17 in the vacuoles. The remaining soybean PPOs were mainly located in chloroplasts (Table 1 and Table 2). In the wild soybean, GsPPO3 and GsPPO5 were predominantly located in the nucleus, GsPPO14 in the cytoplasm, and GsPPO15 in vacuoles, while the remaining PPOs were mainly localized in chloroplasts.

Overall, GmPPO2, GmPPO10, GmPPO11, GsPPO1, GsPPO9, and GsPPO10 showed minimal differences in terms of amino acid length, relative molecular mass, isoelectric point, and predicted subcellular localization (Table 1 and Table 2). In contrast, the other PPO proteins exhibited varying degrees of evolution or degradation, leading to functional differences among these proteins.

### 3.2. Analysis of Conserved Domains and Gene Structure

**Figure 1:**
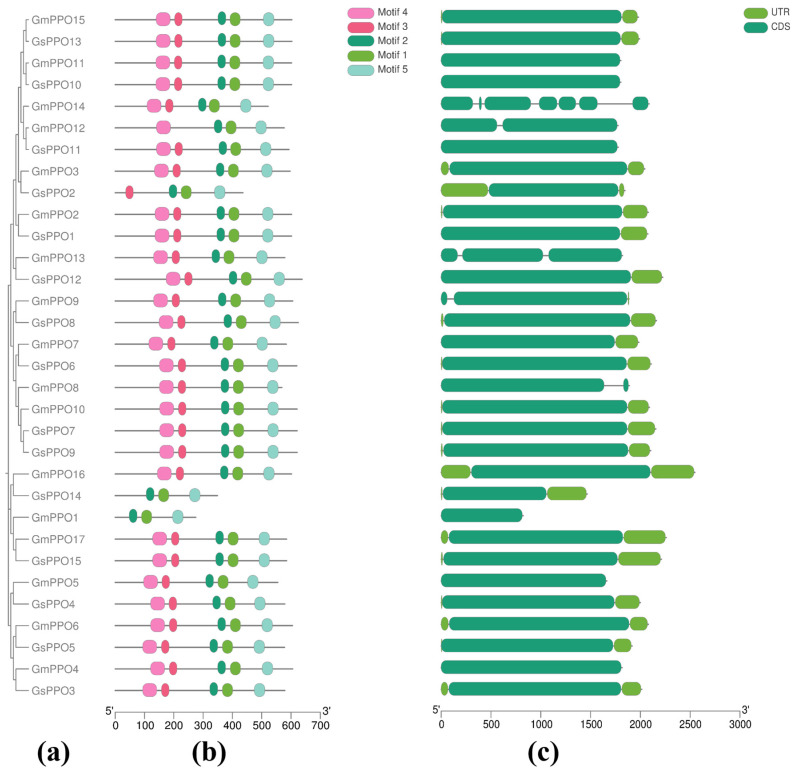
Gene structure and conserved motifs of *GmPPOs* and *GsPPOs*. (**a**) Evolutionary tree. (**b**) Distribution of conserved motifs on genes. (**c**) Gene structure.

To further explore the motifs of the *PPO* gene family, the full-length protein sequences of the family members were analyzed using the MEME tool. A total of five conserved motifs were identified and designated as motifs 1–5 (Figure 1b, Table S1). Among them, motifs 1, 2, and 5 were common to all the *PPO* family members, representing the core motifs of the *PPO* gene family. Notably, *GmPPO1* and *GsPPO14* contained the fewest motifs in the soybean and wild soybean, respectively, suggesting that the functions of these two genes may differ significantly from those of other family members. Additionally, no significant differences in conserved motifs were observed between the soybean and wild soybean genes. These variations in motif arrangement appeared to contribute to the diversity in sequence structure among PPOs, indicating that they may play different roles in plant development.

A gene structure analysis revealed that most PPO genes in both the soybean and wild soybean had a single CDS, with only five soybean *PPO* genes containing two or more CDSs (*GmPPO8*, *GmPPO9*, *GmPPO12*, *GmPPO13*, and *GmPPO14*). In the wild soybean genes, only one CDS was present (Figure 1c). Furthermore, the 5′UTR was either absent or very short for most genes. However, *GsPPO2* and *GmPPO16* contained longer 5′UTR sequences, which could impact the function of the entire gene family, since the regulation of 5′UTR-mediated translation initiation plays an important role in gene regulation [3,30].

### 3.3. Phylogenetic Analysis of PPO Gene Families

**Figure 2:**
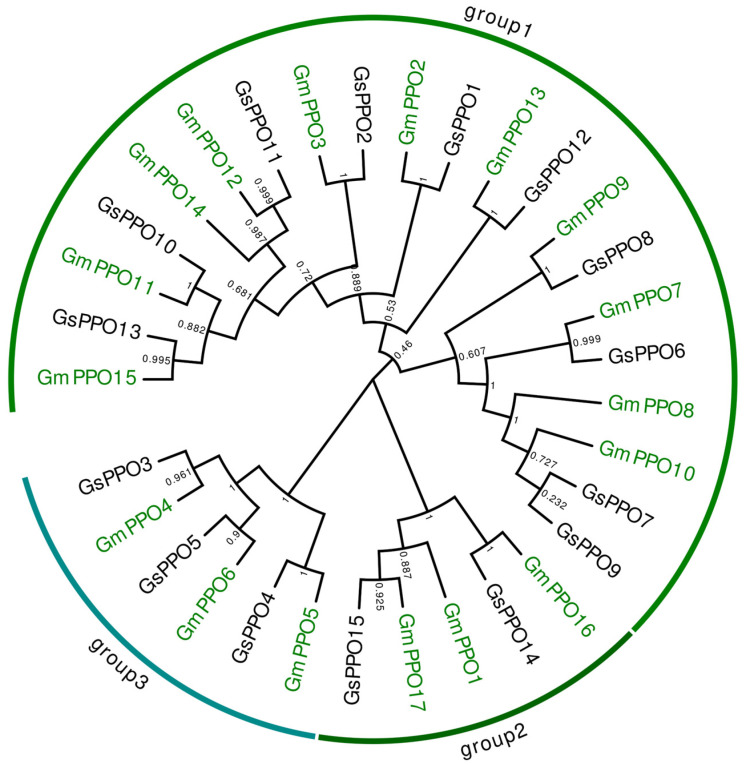
Phylogenetic tree of the *PPO* gene family in the soybean and wild soybean.

To further understand the phylogenetic relationship among the different *PPOs*, their protein sequences were compared via the MeGA 7 software, and a phylogenetic tree was constructed (Figure 2). In total, 32 *PPO* gene family members could be divided into three groups with 21, 5, and 6 members each. The soybean and wild soybean genes were almost evenly distributed, indicating that *GmPPOs* were indeed domesticated from *GsPPOs*, sharing high similarity.

### 3.4. Chromosomal Location and Gene Duplication Analysis of PPO Genes

**Figure 3:**
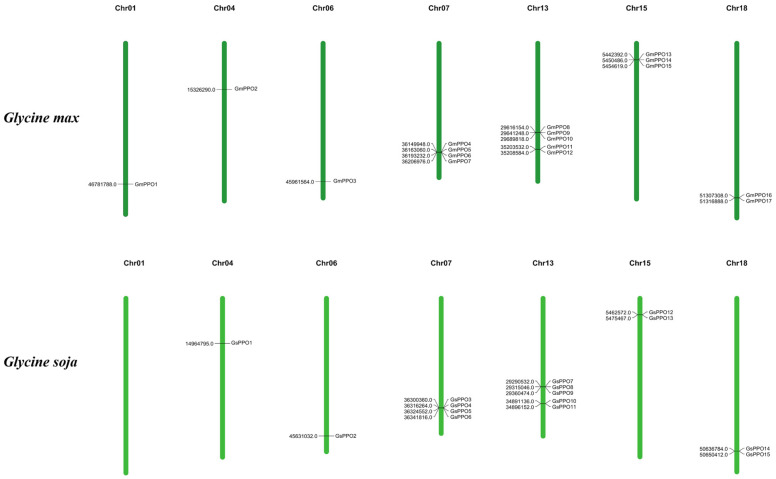
Chromosomal localization of the *PPO* gene family in the soybean and wild soybean.

Information regarding the chromosomal location of each *PPO* gene in the soybean and wild soybean was extracted from the GFF3 files, and a chromosome distribution map was prepared (Figure 3). The results showed that these genes were mainly concentrated on Chr07, Chr13, and Chr15. There was no *PPO* gene on wild soybean Chr01, but during domestication, a *PPO* gene was added to Chr01. The findings suggested that this gene may play an important role in some growth and development processes of soybeans. Similarly, *GmPPO14* was added to Chr15 after domestication. Nevertheless, given the large fragment replication in the expanded region of *GmPPO14*, we speculated that its expansion may not have a significant effect.

**Figure 4:**
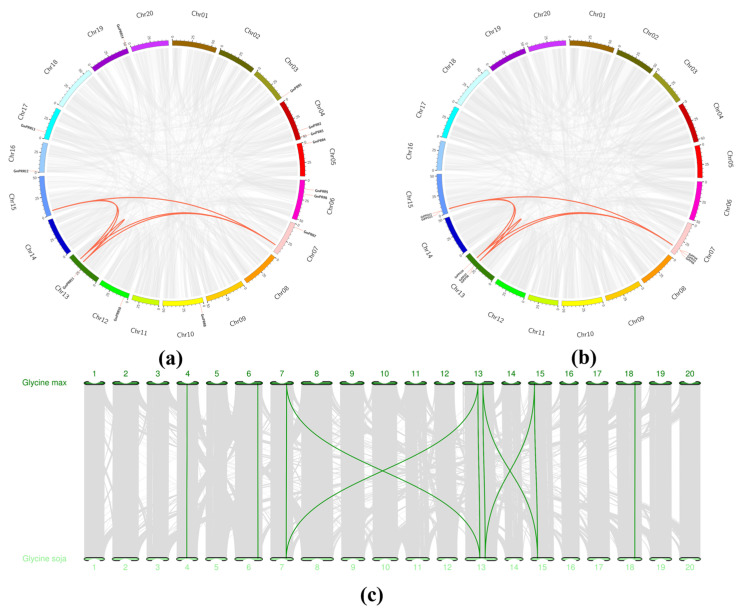
Collinearity analysis within and between soybean and wild soybean. (**a**) Collinearity analysis within soybean. (**b**) Collinearity analysis within wild soybean. (**c**) Collinearity analysis between soybean and wild soybean. The numbers in the figure represent chromosome numbers.

MCScanX was employed to analyze the collinearity of genomic data, and the duplicated genes from the *PPO* gene family were identified to prepare a collinearity map (Figure 4a,b). In total, 7 and 8 pairs of genes with fragment repeats were found in the soybean and wild soybean, respectively, mainly concentrated on Chr07, Chr13, and Chr15 (Table S2).

To further understand the phylogenetic mechanisms of the *PPO* gene family in the soybean and wild soybean, evolutionary homology was analyzed through comparative genomics (Figure 4c). In the figure, gray lines represent the collinear regions of different chromosomes, and green lines represent the collinearity relationship. As mentioned above, there were a few duplicated genes in the soybean and wild soybean (Figure 4a,b). However, 75 pairs of duplicated genes were identified between these species (Table S2), and gene replication was detected across most chromosomes (Figure 4c). This indicates that the entire *PPO* gene family was mainly produced via gene replication, and that gene replication played an important role in driving the evolution of *PPOs*.

**Figure 5:**
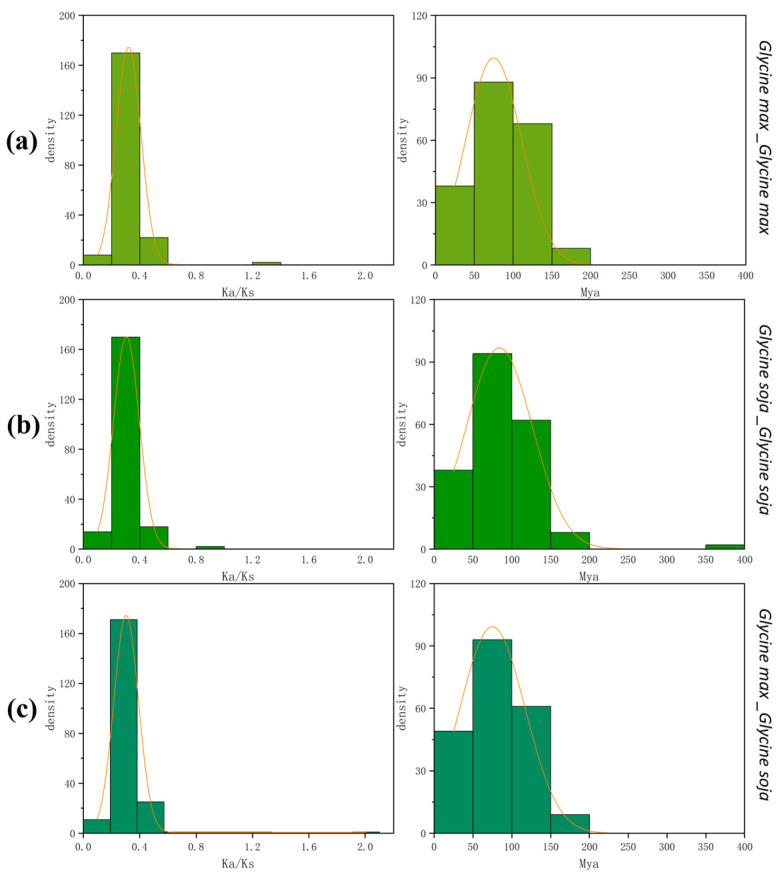
Ka/Ks and Mya analysis within and between different soybean species. (**a**) Ka/Ks and Mya analysis within soybean. (**b**) Ka/Ks and Mya analysis within wild soybean. (**c**) Ka/Ks and Mya analysis between soybean and wild soybean.

In addition, the Ka/Ks analysis within the soybean (Figure 5a), within the wild soybean (Figure 5b), and between the soybean and wild soybean (Figure 5c) showed that *PPOs* were mainly subjected to purifying selection, which was conducive to maintaining the stability of *PPO* gene family members in soybean. This selection also facilitated evolution and adaptation to environmental changes in soybeans. However, the ratio of *GmPPO9* to *GsPPO8* and *GmPPO4* to *GsPPO5* was greater than one, indicating that these two gene pairs were positively selected during evolution and evolved rapidly in recent times, providing valuable insights for research into soybean domestication. Previous studies have shown that soybeans have undergone two whole genome duplications (WGDs) at 59 and 5–13 Mya [31,32,33,34], respectively. This was consistent with the timing of gene duplication in the *PPO* gene family. Additionally, more gene duplication occurred between 50 and 100 Mya (Figure 5) [35]. Due to WGD, the cis-acting element pattern underwent remodeling [36], affecting the transcriptional differentiation of the duplicated soybean genes. Therefore, we also analyzed the cis-acting elements in the promoter regions of these genes [37].

### 3.5. Analysis of the Cis-Acting Elements of the PPO Gene Family

**Figure 6:**
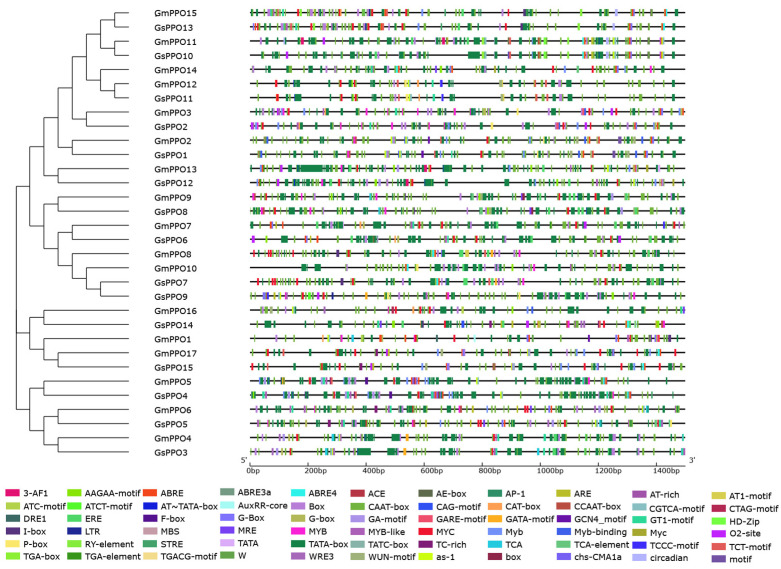
Prediction analysis of the cis-acting elements in the soybean and wild soybean.

Given that WGDs are often accompanied by changes in cis-acting elements, to compare the changes in cis-acting elements in soybean and wild soybean and to further investigate the possible transcriptional regulatory mechanism of *PPO* genes in these species, we analyzed the cis-acting elements in the promoter sequences that were 1500 bp upstream of the 32 *PPO* genes. We identified 66 different cis-acting elements (Figure 6, Table S3), which could be broadly classified into three categories: (i) light response elements (18 types, including ACE, 3-AF1, ACE, AE-box, AT-rich, AT1 motif, ATC motif, ATCT motif, Box, GA motif, GATA motif, I-box, TCCC motif, TCT motif, chs-CMA1a, G-box, MRE, and circadian); (ii) hormone-related elements (16 types, including AAGAA motif, AuxRR core, CGTCA motif, CTAG motif, ERE, GARE motif, P-box, TGA-box, TATC-box, ABRE, ABRE3a, ABRE4, STRE, TCA, TCA element, as-1, and F-box); and (iii) stress response elements (25 types, including DRE1, GT1 motif, GCN4 motif, CAT box, LTR, AP-1, ARE, CAG motif, CCAAT box, MBS, MYB, MYB-like, MYC, Myb, Myb-binding, Myc, TC-rich, TATC-box, W, WRE3, WUN motif, as-1, ABRE, ABRE3a, ABRE4, STRE, and F-box). In addition, a few other developmentally regulated motifs such as the RY element, O2 site, and HD-Zip were also detected. Interestingly, soybean and wild soybean genes within the same branch of the evolutionary tree tended to share a higher similarity with respect to their cis-acting element distribution. Notably, many of the identified cis-acting elements were found to be involved in multiple processes, such as hormone responses, stress responses, and developmental regulation. This distribution of the cis-acting elements suggested that the *PPO* gene family plays a significant role in both the growth and development of soybean and wild soybean, as well as their responses to various types of stress.

### 3.6. Analysis of the Gene Expression of PPO Genes Throughout the Growth Period

**Figure 7:**
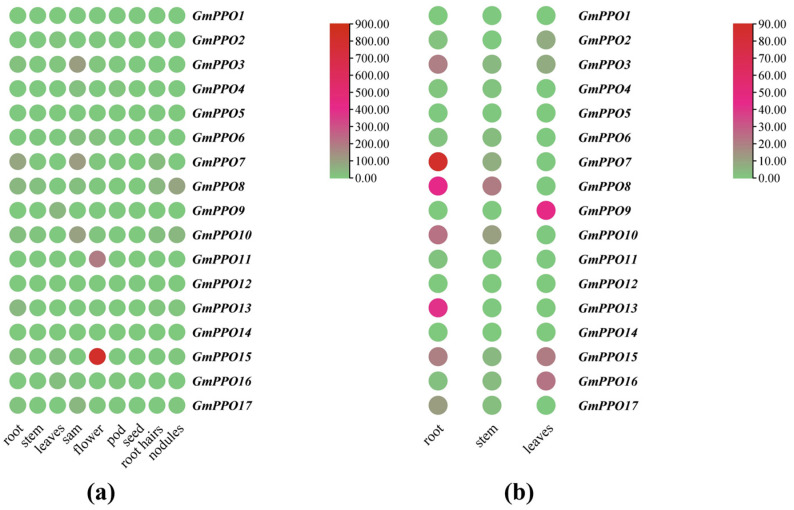
Gene expression in different tissues of soybean plants. (**a**) Gene expression throughout the plant. (**b**) Gene expression in roots, stems, and leaves.

To elucidate the spatial expression patterns of the *PPO* gene family, we obtained soybean gene expression data in various tissues through a public database and visualized the results using TBtools (Figure 7a, Table S4). The analysis revealed distinct tissue specificity among the *GmPPO* genes. For example, *GmPPO15* and *GmPPO11* were highly expressed in flowers, while *GmPPO3*, *GmPPO7*, and *GmPPO10* showed high expression levels in the shoot apical meristem (SAM). However, the gene expression data for wild soybeans was not available in the public database, and the existing soybean data did not reflect changes across different growth stages. To address this, we resampled and analyzed the gene expression in roots, stems, and leaves throughout the entire growth period to obtain a comprehensive dataset for follow-up analyses.

Based on the expression changes across tissues during the growth cycle, we re-mapped the transcriptome data for the roots, stems, and leaves (Figure 7b). This revealed more significant trends in many tissues that were not evident from the previous data. For example, most of the genes in the *PPO* family were found to be expressed in the roots, and their expression was lower in the stems.

**Figure 8:**
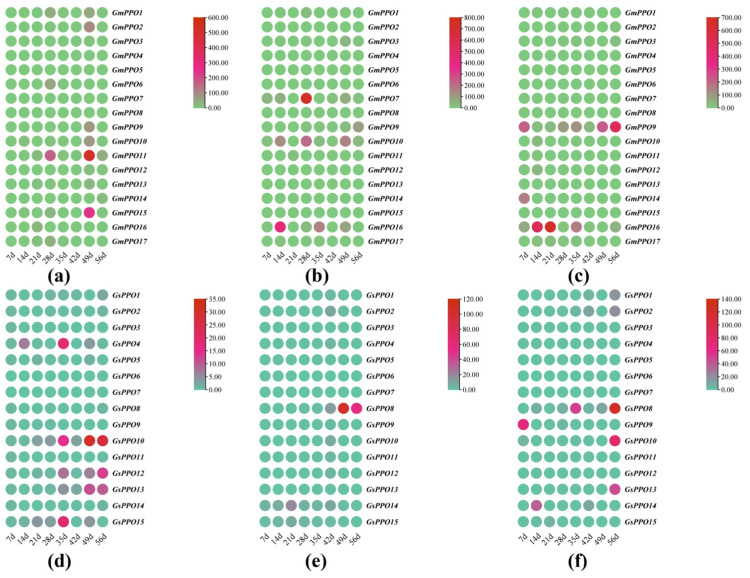
Gene expression in different tissues of soybean and wild soybean plants during the whole growth period. (**a**–**c**) Soybean roots, stems, and leaves. (**d**–**f**) Wild soybean roots, stems, and leaves.

The gene expression patterns of the *PPOs* were observed throughout the growth period (Figure 8). Based on the evolutionary characteristics of wild soybean and soybean genes, we found that *GmPPO11* and *GsPPO10*, as well as *GmPPO15* and *GsPPO13*, exhibited similar expression patterns in the roots (Figure 8a,d). Similarly, *GmPPO9* and *GsPPO8*, along with *GmPPO16* and *GsPPO14*, shared similar expression trends in the stems (Figure 8b,e). *PPO* gene expression was more abundant in the stems of the soybean than in those of the wild soybean, likely due to the evolution of stem traits in domesticated soybeans. Additionally, *GmPPO9* and *GsPPO8*, as well as *GmPPO16* and *GsPPO14*, exhibited similar patterns in the leaves (Figure 8c,f). We speculate that these gene pairs may play an important role in the domestication of stem and leaf traits in soybean plants.

### 3.7. Gene Expression Analysis of PPO Genes Under Salt Stress

**Figure 9:**
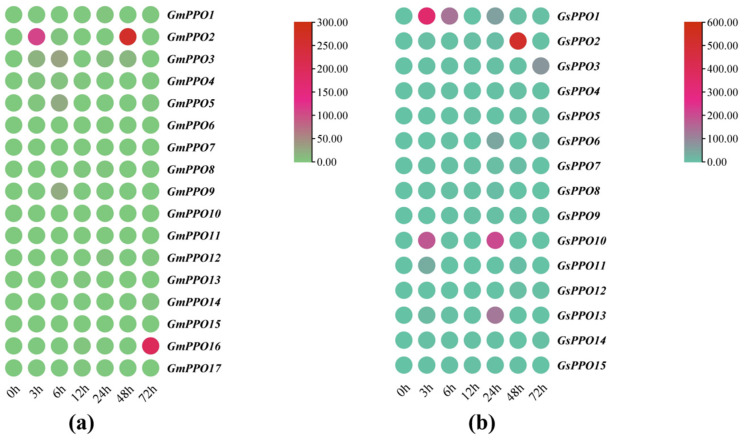
Gene expression in soybean and wild soybean plants under salt stress. (**a**) Gene expression in soybean plants. (**b**) Gene expression in wild soybean plants.

Given that some *PPO* family members contain stress response elements, we further explored their response to salt stress to lay the groundwork for identifying potential salt tolerance genes (Figure 9). The heat map showed that 7 *GsPPO* genes (*GsPPO1*, *GsPPO2*, *GsPPO3*, *GsPPO6*, *GsPPO10*, *GsPPO11*, and *GsPPO13*) exhibited significant upregulation in response to salt stress (Figure 9b), while 5 *GmPPO* genes (*GmPPO2*, *GmPPO3*, *GmPPO5*, *GmPPO9*, and *GmPPO16*) showed a similar trend (Figure 9a). Among these genes, *GmPPO16* reached its peak expression after 72 h of salt stress, while *GsPPO2* expression peaked at 48 h of treatment. Both *GmPPO2* and *GsPPO1* showed significant responses within 3 h of salt stress, suggesting that these duplicated genes may play key roles in the salt tolerance of soybeans.

### 3.8. Prediction of 3D Structure and Protein Interactions of PPO Gene Family Members in Soybean and Wild Soybean

**Figure 10:**
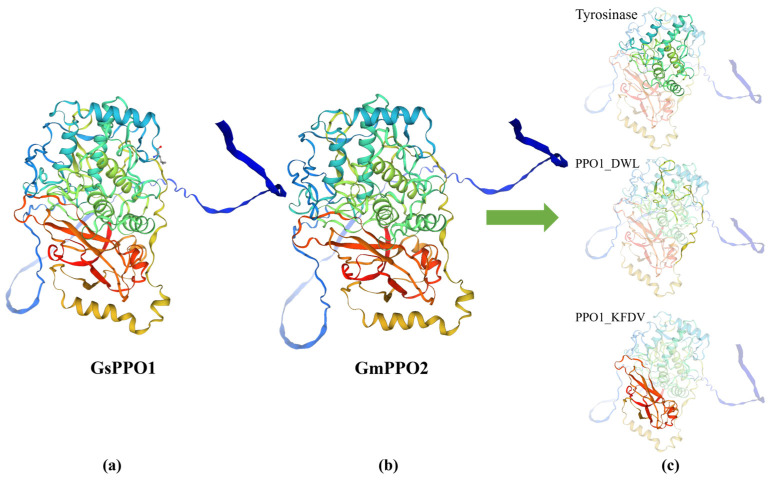
Comparison of the 3D structures of *GmPPO2* and *GsPPO1*. (**a**) *GsPPO1*. (**b**) *GmPPO2*. (**c**) Domain details of the 3D structure diagram. The blue-green indicates the Tyrosinas domain, the yellow-green indicates the PPO1_DWL domain, and the red indicates the PPO1_KFDV domain.

**Figure 11:**
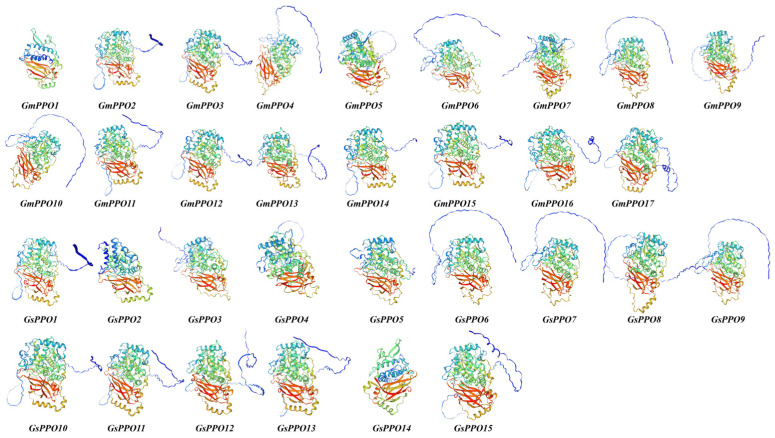
Three-dimensional structure diagrams of various members of the soybean and wild soybean *PPO* gene family. The blue-green indicates the Tyrosinas domain, the yellow-green indicates the PPO1_DWL domain, and the red indicates the PPO1_KFDV domain.

Building on the salt stress experiment results involving the *GmPPO2* and *GsPPO1* gene pairs, we predicted their 3D structures (Figure 10). The analysis revealed a high degree of structural similarity between these two genes. Using CLUSTAL multiple sequence alignment, we found that the CDSs of *GmPPO2* and *GsPPO1* differ by only three bases, which does not affect translation, resulting in identical protein sequences (File S1). This structural similarity is consistent with their similar expression patterns under salt stress. This suggests that these genes share functional characteristics and likely participate in similar regulatory pathways in response to salt stress. In addition to *GmPPO2* and *GsPPO1*, we also predicted the 3D structures of the remaining 16 *GmPPOs* and 14 *GsPPOs* (Figure 11). The results showed that the 3D structures were consistent with the phylogenetic tree results, such that genes closer on the phylogenetic tree shared more similar 3D structures. Additionally, all the 3D protein structures contained an obvious Tyrosinase domain, a less prominent PPO1_DWL domain located within the upper-right region of the protein, and an evident PPO1_KFDV domain. Collectively, the results showed that PPOs may also have similar functions.

**Figure 12:**
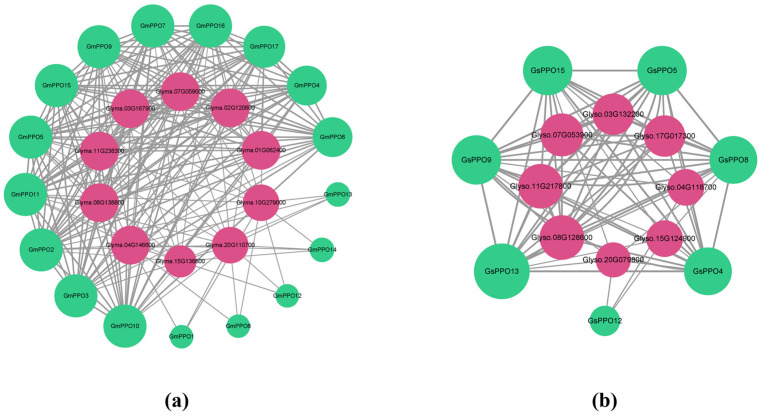
Protein–protein interaction network diagram. (**a**) Protein interaction network of soybean. (**b**) Protein interaction network of wild soybean. The green circles represent members of the PPO family, the red circles represent interactors, and the thickness of the lines indicates the combined score.

Therefore, we used the STRING online tool to predict the protein–protein interactions for the *PPO* gene family members. Meanwhile, homologous gene mapping results from OrthoVenn were also integrated. Accordingly, the protein interaction network was obtained (Figure 12). Surprisingly, the protein interaction network of the soybean was significantly richer than that of the wild soybean. In the soybean network, all the *PPO* genes were involved, with close interactions between them. In contrast, in the wild soybean, only 7 *PPO* genes (*GsPPO4*, *GsPPO5*, *GsPPO8*, *GsPPO9*, *GsPPO12*, *GsPPO13*, and *GsPPO15*) were involved. Interestingly, these *GsPPOs* were the ones that showed high specificity in expression across different tissues throughout the growth period.

Accordingly, we found that the interacting proteins of *GmPPO2* were primarily involved in metabolism and protein dephosphorylation, suggesting that *GmPPO2* may play a significant role in salt stress resistance (Table S6, Figure S2). To explore this hypothesis, we used AlphaFold 3 and predicted the protein interactions of *GmPPO2* within the STRING network (Table 3). The top-ranked interactions were again associated with metabolism and protein dephosphorylation. Based on these interactions, we speculated that the genes *Glyma.07G059000*, *Glyma.10G279000*, and *Glyma.03G167900* could be potential candidates involved in the salt stress response mediated by *GmPPO2*.

## 4. Discussion

A large number of *PPO* gene family members have been identified and cloned in plants [38,39,40], but relatively few studies have focused on the *PPO* genes in soybeans and wild soybeans. Previous studies have identified 11 members in the *GmPPO* family [15], but the current study identified 17 genes in this family (Table 1), likely due to the differences in soybean genome versions and incomplete genome data (Table S7). Based on previous searches and identifications, we added six new members of this family each to Chr01, Chr04, Chr13, Chr15, and Chr18. Therefore, we recommend renaming these genes according to their chromosomal order. Additionally, we identified *PPO* gene family members in wild soybeans for the first time (Table 2), revealing that wild soybeans share considerable similarities with soybeans in terms of *PPO* family members.

Most studies have suggested that the PPO gene has two Cu-containing binding regions (Cu A and Cu B), which is consistent with the structure of our motif, although it has also been suggested that there may be a third Cu-containing region. We did not find a description of motif4, but judging from the motifs, most gene family members have a complete set of five motifs, including the unknown motif4, so we hypothesize that motif4 may be a Cu C-binding region [14,41,42]. With regard to gene structure, we found that most *PPO* genes, in both the soybean and wild soybean, lack introns. This is consistent with most *PPO* genes in dicotyledonous plants, which typically do not contain introns. In contrast, the *PPO* genes in monocotyledonous plants generally contain introns [15]. Although introns are removed during transcription, they are thought to play an important role in plant evolution by contributing to the acquisition of new gene functions [43]. Specifically, there were five genes in the soybean with more introns, indicating that these five genes may have new functions, different from those in the wild soybean. Therefore, this difference between soybeans and wild soybeans could explain the evolution of the *PPO* gene family during soybean domestication.

According to previous research, the ancestor of *PPO* genes in monocots also lacked introns. However, with the domestication of soybeans, introns were acquired, indicating that the *PPO* gene family contains dynamic adaptive genes [44]. This dynamic change is also reflected in the domestication mechanisms of soybeans. *PPO* genes are not present in Chr01 of wild soybeans, but an additional *PPO* gene was detected in Chr01 in soybeans (Figure 3). Some researchers have stated that the absence of *PPO* genes in *Arabidopsis* indicates that these genes are not essential for metabolic function or demonstrate functional redundancy. In *Arabidopsis*, the absence of *PPO* genes may be compensated for by other oxidases [45]. Soybeans are less stress tolerant than wild soybeans, and the *GmPPO1* gene is involved in the stress tolerance process [6,7]. On the other hand, although soybeans were domesticated from wild soybeans, and the genetic resources of wild soybeans should be much richer than those in soybeans, but from our co-collinearity analyses of the soybean and wild soybean (File S1), *GmPPO1* does not exist in co-collinearity with the *GmPPO1* genes in the wild soybean, and as for the co-collinearity of *GmPPO1* with other genes, I examined all the gene pairs between the soybean and wild soybean obtained by using OrthoVenn3. All the gene pairs obtained for the soybean and wild soybean did not find the presence of *GmPPO1*. Kim et al. suggested that more nucleic acids and proteins are present in soybeans than in wild soybeans, so a large number of genes related to hydrolase and transferase activities were added or lost in soybeans [46]. Therefore, we speculate that *GmPPO1* is related to transferases and is a product of the domestication of soybeans, with newly added genes during the domestication process. However, more evidence is needed to confirm this idea through our later studies or those of other scientists.

From a phylogenetic perspective, both soybeans and wild soybeans contain large *PPO* gene families, similar to the *PPO* gene family in poplar [47]. This large gene family size may be attributed to gene duplication events [15]. Our analysis also revealed that the *PPO* gene family underwent multiple duplication events from wild soybeans to domesticated soybeans (Figure 4c). A genome-wide study comparing orthologous gene sets from *Arabidopsis*, poplar, rice, and *Physcomitrella patens* found that genes with stress-responsive expression patterns (such as those involved in defense) are more likely to undergo lineage-specific tandem duplications than genes involved in primary metabolic and cellular functions [48]. Thus, the tandem gene duplications in the *PPO* gene family are likely related to stress responses and ecological adaptations.

Gene duplication events are often associated with changes in the distribution of cis-acting elements. Therefore, we analyzed the cis-acting elements of the *PPO* genes in the soybean and wild soybean and found a high degree of consistency in the types of elements present, in line with the functional roles of the *PPO* family members, including stress and light responses. This provides a strong reference for further studies on the functional roles of *PPOs*.

Therefore, we analyzed the expression patterns of the *PPO* genes in both the soybean and wild soybean and found that duplicated gene pairs also showed high similarity in expression patterns. For example, *GmPPO9* and *GsPPO8*, and *GmPPO16* and *GsPPO14*, showed highly similar expression patterns in the stems and leaves. Meanwhile, *GmPPO2* and *GsPPO1* also showed high similarity in expression patterns under salt stress. These results, along with the 3D structure predictions, further highlight that gene duplication events contribute to functional similarities among these gene family members. As the *PPO* gene family was transferred from wild soybeans to soybeans, these duplication events made the functions of the gene family members highly similar, and the genes were retained during the domestication process. This indicated that the genes played a crucial role in the adaptability, growth, and development of domesticated soybeans.

In addition, the analysis of the protein interaction networks of the *PPO* genes in the soybean and wild soybean revealed that the interaction network of soybeans was significantly more complex than that of wild soybeans (Figure 12). This difference may be related to the more extensive exploration of the soybean gene regulatory network. The gene interaction network of wild soybeans has not been studied extensively, even though the genome of wild soybeans is a natural treasure. Given the potential for discovering genes and regulatory pathways related to soybean domestication, we encourage more research into the gene interaction regulatory networks of wild soybeans.

In particular, the *GmPPO2* and *GsPPO1* gene pair showed remarkable consistency across various aspects—gene structure, 3D structure, expression patterns, and evolutionary relationships. To further investigate this, we conducted protein interaction predictions and identified three potential candidate genes involved in the salt stress regulatory network. In the future, we hope to validate these findings through additional research (Figure 10). Collectively, in the present study, we compared and analyzed the *PPOs* in soybeans and wild soybeans to provide a reference for the subsequent functional analysis of *PPOs* and to further elucidate the domestication process of soybeans, which can provide a reference for the creation of new soybean varieties.

## Figures and Tables

**Table 1:** Basic information and subcellular localization of GmPPO proteins.

Name	Gene ID	Amino Acid Number	MW (Da)	PI	Subcellular Localization Score			
					Cytoplasm	Nucleus	Chloroplast	Vacuole
GmPPO1	*Glyma.01G139800*	274	31,230.1	5.84	5	4	3	
GmPPO2	*Glyma.04G121700*	601	67,204.5	7.12			14	
GmPPO3	*Glyma.06G270400*	596	66,907.4	7.79		1	12	
GmPPO4	*Glyma.07G193300*	605	68,939.9	6.18		2	8	
GmPPO5	*Glyma.07G193400*	554	63,047	6.85		4	5	
GmPPO6	*Glyma.07G193500*	604	68,829.9	6.27		1	9	
GmPPO7	*Glyma.07G193600*	583	66,247.4	6.22	1	6	5	
GmPPO8	*Glyma.13G183000*	568	63,936.1	7.76		2	10	
GmPPO9	*Glyma.13G183200*	605	68,257.8	7.86	3	1	8	
GmPPO10	*Glyma.13G183500*	620	69,586.2	6.29		2	9	
GmPPO11	*Glyma.13G242300*	601	67,723.9	7.27		1	10.5	
GmPPO12	*Glyma.13G242400*	576	64,359.9	7.36		1	12	
GmPPO13	*Glyma.15G071000*	578	64,686.8	7.44			12	
GmPPO14	*Glyma.15G071100*	521	59,024.3	8.68	1	2	7	
GmPPO15	*Glyma.15G071200*	602	67,819.2	7.67		1	12	
GmPPO16	*Glyma.18G225000*	601	68,922.9	7.39	1	1	2	7
GmPPO17	*Glyma.18G225100*	584	66,815.1	6.55	1		1	6

Note: The numbers in the subcellular localization column represent the likelihood of the protein being located in that particular subcellular compartment. The higher the number, the greater the probability that the protein is localized to that specific subcellular compartment.

**Table 2:** Basic information and subcellular localization of GsPPO proteins.

Name	Gene ID	Amino Acid Number	MW (Da)	PI	Subcellular Localization Score				
					Cytoplasm	Nucleus	Chloroplast	Peroxisome	Mitochondrion
GsPPO1	*Glyso.04G102600*	602	67,204.5	7.12			14		
GsPPO2	*Glyso.06G232000*	436	49,464.6	6.38	2	3	3	3	3
GsPPO3	*Glyso.07G158900*	579	66,147.8	6.12	4	6	2		2
GsPPO4	*Glyso.07G159000*	579	65,503.7	6.88		1	11	1	1
GsPPO5	*Glyso.07G159100*	578	66,037.8	6.21	3	6	2		2
GsPPO6	*Glyso.07G159200*	620	69,897.5	6.34			12.5		
GsPPO7	*Glyso.13G146200*	621	69,572.1	6.29		2	9	1	1
GsPPO8	*Glyso.13G146400*	625	70,134.9	8.16			12.5		
GsPPO9	*Glyso.13G146700*	621	69,600.2	6.29		2	9	1	1
GsPPO10	*Glyso.13G200100*	602	67,751.9	7.27			10.5	1	
GsPPO11	*Glyso.13G200200*	593	66,072.9	7.35		1	12	1	
GsPPO12	*Glyso.15G066400*	638	71,107	7.18			12.5		1.5
GsPPO13	*Glyso.15G066500*	603	67,819.2	7.67		1	12	1	
GsPPO14	*Glyso.18G171400*	349	39,845.6	5.6	8	3	2		
GsPPO15	*Glyso.18G171500*	585	66,829.2	6.62					

Note: The numbers in the subcellular localization column represent the likelihood of the protein being located in that particular subcellular compartment. The higher the number, the greater the probability that the protein is localized to that specific subcellular compartment.

**Table 3:** Functional annotation and interaction prediction scores of GmPPO2 interacting proteins.

ID	Functional Annotation	Chain Pair pae min	Chain ptm	Score
Glyma.08G138800	Reproduction	0.76, 26.45; 27.5, 0.76	0.79, 0.82	26.45
Glyma.07G059000	Organic acid metabolic process	0.76, 26.95; 26.32, 0.76	0.76, 0.88	26.32
Glyma.11G238300	Reproduction	0.76, 26.28; 26.8, 0.76	0.79, 0.81	26.28
Glyma.10G279000	Protein dephosphorylation	0.76, 26.77; 26.21, 0.76	0.78, 0.48	26.21
Glyma.03G167900	Organic acid metabolic process	0.76, 25.57; 26.38, 0.76	0.78, 0.89	25.57
GmPPO6	-	0.76, 24.31; 23.84, 0.76	0.73, 0.7	23.84
GmPPO17	-	0.76, 22.59; 22.89, 0.76	0.72, 0.74	22.59
GmPPO7	-	0.76, 22.5; 22.44, 0.76	0.73, 0.75	22.44
GmPPO5	-	0.76, 23.27; 22.18, 0.76	0.73, 0.73	22.18
GmPPO10	-	0.76, 22.57; 21.94, 0.76	0.74, 0.71	21.94
Glyma.20G110700	Protein dephosphorylation	0.76, 25.05; 21.54, 0.76	0.76, 0.84	21.54
GmPPO9	-	0.76, 21.38; 22.98, 0.76	0.72, 0.74	21.38
GmPPO4	-	0.76, 24.36; 24.01, 0.76	0.71, 0.68	21.01
GmPPO16	-	0.76, 21.92; 19.99, 0.76	0.73, 0.73	19.99
Glyma.02G120600	Nitrogen compound metabolic process	0.76, 16.53; 16.66, 0.76	0.76, 0.89	16.53
Glyma.04G146600	Double-strand break repair via homologous recombination	0.76, 15.82; 17.74, 0.76	0.78, 0.85	15.82
GmPPO15	-	0.76, 14.39; 14.92, 0.76	0.61, 0.61	14.39
Glyma.15G136600	Tyrosinase copper-binding domain, and O-acyltransferase WSD1, C-terminal	0.76, 14.39; 13.92, 0.76	0.61, 0.61	13.92
GmPPO3	-	0.76, 8.12; 7.72, 0.76	0.78, 0.78	7.72
Glyma.01G062400	Nitrogen compound metabolic process	0.76, 6.73; 8.87, 0.76	0.78, 0.9	6.73
GmPPO11	-	0.76, 4.34; 4.31, 0.76	0.77, 0.77	4.31

## Data Availability

Further inquiries on data resources can be directed to the first author.

## Supplementary Materials

The following supporting information can be downloaded at https://www.mdpi.com/article/10.3390/genes16010017/s1: File S1: Script file and multi-sequence alignment results; File S2: Table S1: Name and sequence of the conserved motifs; Table S2: Intra- and inter-species covariance of gene pairs in soybeans and wild soybeans; Table S3: Information on cis-acting elements (Excel Table S3); Table S4: Phtozome database transcriptome raw data; Table S5: List of primers used for qRT-PCR; Table S6: Summary of interacting proteins (Excel Table S6); Table S7: Gene Nomenclature Control List; Figure S1. Motif visualization results; Figure S2. Protein–protein interaction network diagram of GmPPO2.
