# Supplementary material for: Genome-Wide Identification and Comprehensive Analysis of the PPO Gene Family in Glycine max and Glycine soja
Source: Genes (Basel). 2024 Dec 26;16(1):17. doi: 10.3390/genes16010017 (PMC11764901; doi:10.3390/genes16010017)
Supplement: Supplementary file 1 [file genes-16-00017-s001.zip › File S1.docx]

**The perl script used in the text is as follows:**

use Data::Dumper;
use Getopt::Long;
use strict;
use Cwd qw(abs_path getcwd);


my %opts;

GetOptions (\%opts,"id=s","tandem=s","od=s","name=s"); 

my $od=$opts{od};
$od||=getcwd;
$od=abs_path($od);
unless(-d $od){    mkdir $od;}

*####get target gene id*

my $gene;
my @info;
my %hashG;
open (IN,"$opts{id}") || die "open $opts{id} failed\n";
while(<IN>){
    chomp;
    @info=split(/\s+/,$_);
    $gene=$info[0];
    $hashG{$gene}=$gene;
}
close(IN);


*#######select tandem*

my $Agene;
my $Bgene;
open(OUT,">$od/$opts{name}.tandem")||die "open $od/$opts{name}.tandem failed\n";
open (IN,"$opts{tandem}") || die "open $opts{tandem} failed\n";
while(<IN>){
    chomp;
    @info=split(/,/,$_);
    $Agene=$info[0];
    $Bgene=$info[1];
    if(exists $hashG{$Agene} && exists $hashG{$Bgene}){
        print OUT $Agene."\t".$Bgene."\n";
    }

}
close(IN);
close(OUT);

**CLUSTAL multiple sequence alignment:**

GmPPO2 ATGGCTTCAATCTCTCCTATATCCTTCGTGAGTGCAATCAATAATGTCTCTTCCAACTCA 60

GsPPO1 ATGGCTTCAATCTCTCCTATATCCTTCGTGAGTGCAATCAATAATGTCTCTTCCAACTCA 60

GmPPO2 TCCAATTCCCCTTCTTCCTTGCATCATCCCTTTTCACAAATTCAATCCGCTAAATATCGA 120

GsPPO1 TCCAATTCCCCTTCTTCCTTGCATCATCCCTTTTCACAAATTCAATCCGCTAAATATCGA 120

GmPPO2 AAACCAAAACGCCATCATCATATTCCTAGAGTGACATGCAGTGACAACCAAAAACCAAAC 180

GsPPO1 AAACCAAAACGCCATCATCATATTCCTAGAGTGACATGCAGTGACAACCAAAAACCAAAC 180

GmPPO2 ACATCTGGAGAACTAGTACTCCCACATAGGAGGAACATTCTACTTGGCCTAGGAGGGCTT 240

GsPPO1 ACATCTGGAGAACTAGTACTCCCACATAGGAGGAACATTCTACTTGGCCTAGGAGGGCTT 240

GmPPO2 TGTGGTGCTGCTGCTACTCTTAACAACATCCCTTTTGCCAATGCTGCCCCAATACTTGGT 300

GsPPO1 TGTGGTGCTGCTGCTACTCTTAACAACATCCCTTTTGCCAATGCTGCCCCAATACTTGGT 300

GmPPO2 CCAGACCTAACCACATGTGTTCAAGCAGAACTACCCGAAGGTGTAGAACCCACCAATTGT 360

GsPPO1 CCAGACCTAACCACATGTGTTCAAGCAGAACTACCCGAAGGTGTAGAACCCACCAATTGT 360

GmPPO2 TGTCCCCCAATTTCCACAAACATCATAGATTTCAAGTTCCCTCCCTCCAACCAACCCTTG 420

GsPPO1 TGTCCCCCAATTTCCACAAACATCATAGATTTCAAGTTCCCTCCCTCCAACCAACCCTTG 420

GmPPO2 CGTGTACGATCCGCTGCTCATCTGGTCAACAAAGACTATCTAGCTAAATACGAGAAAGCC 480

GsPPO1 CGTGTACGATCCGCTGCTCATCTGGTCAACAAAGACTATCTAGCTAAATACGAGAAAGCC 480

GmPPO2 GTTAACCTGATGAAAAATCTCCCGTCAGATGATCCACGTAGTTTCGCGCAACAAGCCAAA 540

GsPPO1 GTTAACCTGATGAAAAATCTCCCGTCAGATGATCCACGTAGTTTCGCGCAACAAGCCAAA 540

GmPPO2 GTTCATTGTGCTTATTGCGACGGTGGATATCACCAACTAGGCTTCCCTGACCTTGATCTC 600

GsPPO1 GTTCATTGTGCTTATTGCGACGGTGGATATCACCAACTAGGCTTCCCTGACCTTGATCTC 600

GmPPO2 GAAGTGCACTTCTCTTGGCTCTTCTTTCCTTACCACAGATGGTATCTCTATTTCCATGAA 660

GsPPO1 GAAGTGCACTTCTCTTGGCTCTTCTTTCCTTACCACAGATGGTATCTCTATTTCCATGAA 660

GmPPO2 AGGATATTGGCGAGCTTGATCAATGATCCAACCTTTGCTCTTCCATTTTGGAACTGGGAT 720

GsPPO1 AGGATATTGGCGAGCTTGATCAATGATCCAACCTTTGCTCTTCCATTTTGGAACTGGGAT 720

GmPPO2 GCTCCTGGGGGCATGCAACTTCCTTCCATGTACGCAGATCCCAAATCACCCCTTTATGAT 780

GsPPO1 GCTCCTGGGGGCATGCAGCTTCCTTCCATGTACGCAGATCCCAAATCACCCCTTTATGAT 780

GmPPO2 TCTCTACGCAATGCCAACCATCAACCACCAACACTTGTAAACCTTGACTTTACTATCGAG 840

GsPPO1 TCTCTACGCAATGCCAACCATCAACCACCAACACTTGTAAACCTTGACTTTACTATCGAG 840

GmPPO2 GATCCTAATGCAGAGGCAAATATCTCCACCAACCTCACCACAATGTATAGGCAGCTTGTG 900

GsPPO1 GATCCTAATGCAGAGGCAAATATCTCCACCAACCTCACCACAATGTATAGGCAGCTTGTG 900

GmPPO2 TCTAACGCAAAGACTCCAACATTGTTCTTCGGAAATCCTTATCGTGCTGGGGATCAGCCT 960

GsPPO1 TCTAACGCAAAGACTCCAACATTGTTCTTCGGAAATCCTTATCGTGCTGGGGATCAGCCT 960

GmPPO2 AACCCTGGTGGTGGCTCCGTAGAGAGCACTCCACATGGTCCTGTTCATGCATGGACCGGT 1020

GsPPO1 AACCCTGGTGGTGGCTCCGTAGAGAGCACTCCACATGGTCCTGTTCATGCATGGACCGGT 1020

GmPPO2 GATATCAACCACCCTACAATGGAGGACATGGGGAATTTATATGCAGCTGCAAGAGACCCC 1080

GsPPO1 GATATCAACCACCCTACAATGGAGGACATGGGGAATTTATATGCTGCTGCAAGAGACCCC 1080

GmPPO2 ATTTTCTATTGCCACCATTCCAATGTTGATAGGATGTGGTCCATATGGAAAACACTTGGT 1140

GsPPO1 ATTTTCTATTGCCACCATTCCAATGTTGATAGGATGTGGTCCATATGGAAAACACTTGGT 1140

GmPPO2 GGGAAAAGAAGGGATTTAACAGACCCGGATTGGTTAGAATCCGCGTTTCTCTTCTACGAT 1200

GsPPO1 GGGAAAAGAAGGGATTTAACAGACCCGGATTGGTTAGAATCCGCGTTTCTCTTCTACGAT 1200

GmPPO2 GAGAATAAGAACCTTGTGCGTGTGAAGACTAAGGATTGTCTTGACACGAGAAAGTTAGGG 1260

GsPPO1 GAGAATAAGAACCTTGTGCGTGTGAAGACTAAGGATTGTCTTGACACGAGAAAGTTAGGG 1260

GmPPO2 TATGTTTACCAAGATGTTGACATTCCATGGTTAAAATCTAAGCCTACGCCATTAAGGTCA 1320

GsPPO1 TATGTTTACCAAGATGTTGACATTCCATGGTTAAAATCTAAGCCTACGCCATTAAGGTCA 1320

GmPPO2 AGGGCTCAAAAGGTAGAACTGACACCACTTTTTGGTGGTGTTGCTGCAGCACATGCTGCT 1380

GsPPO1 AGGGCTCAAAAGGTAGAACTGACACCACTTTTTGGTGGTGTTGCTGCAGCACATGCTGCT 1380

GmPPO2 GAGACTTCAAGGAATGTGAAGTTCCCATTGGTGTTGGATTCAGTTGTGAGTACAGTGGTG 1440

GsPPO1 GAGACTTCAAGGAATGTGAAGTTCCCATTGGTGTTGGATTCAGTTGTGAGTACAGTGGTG 1440

GmPPO2 AAGAGGCCAAAGAAGTCTAGGAGCAAAAAGGAGAAGGAAGAGAAGGAGGAGATTCTGGTG 1500

GsPPO1 AAGAGGCCAAAGAAGTCTAGGAGCAAAAAGGAGAAGGAAGAGAAGGAGGAGATTCTGGTG 1500

GmPPO2 GTTGAAGGGATTGAGTTTGAGAGCAGCACAGGTGTGAAGTTTGATGTGTTTATTAATGAT 1560

GsPPO1 GTTGAAGGGATTGAGTTTGAGAGCAGCACAGGTGTGAAGTTTGATGTGTTTATTAATGAT 1560

GmPPO2 GAAGATGATAAGTTGGTCAAGCCAGATAATACGGAGTTTGCAGGAAGCTTTGTGAGTGTG 1620

GsPPO1 GAAGATGATAAGTTGGTCAAGCCAGATAATACGGAGTTTGCAGGAAGCTTTGTGAGTGTG 1620

GmPPO2 CCTCATTCGCATGAGCATCACAAAAACAACAAGAAGATTGTTACTTGTTTGAGGTTGGGA 1680

GsPPO1 CCTCATTCGCATGAGCATCACAAAAACAACAAGAAGATTGTTACTTGTTTGAGGTTGGGA 1680

GmPPO2 CTAACGGATTTGTTGGAAGAATTGGGAGCAGAAGATGATGATAGTGTTCTAGTAACATTG 1740

GsPPO1 CTAACGGATTTGTTGGAAGAATTGGGAGCAGAAGATGATGATAGTGTTCTAGTAACATTG 1740

GmPPO2 GTTCCCAAGTATGGGAAAGGGCGAGTTAACATCCGAGGCATCAAGATAGATTTTGTTTCA 1800

GsPPO1 GTTCCCAAGTATGGGAAAGGGCGAGTTAACATCAGAGGCATCAAGATAGATTTTGTTTCA 1800

GmPPO2 GATTGA 1806

GsPPO1 GATTGA 1806
