# Supplementary material for: Genome-Wide Identification and Comprehensive Analysis of the PPO Gene Family in Glycine max and Glycine soja
Source: Genes (Basel). 2024 Dec 26;16(1):17. doi: 10.3390/genes16010017 (PMC11764901; doi:10.3390/genes16010017)
Supplement: Supplementary file 1 [file genes-16-00017-s001.zip › File S2.docx]

**Table S1** Name and sequence of the conserved motifs

| **Code** | **Name** | **Sequence** |
| --- | --- | --- |
| Motif 1 | PPO1_DWL | PDWLESSFLFYDENKNLVRVKVKDCLDTRKLGYVYQDVD |
| Motif 2 | Tyrosinase -Cu B | EDMGTFYSAARDPIFYSHHSNVDRMWSIWKT |
| Motif 3 | Tyrosinase -Cu A | LQVHNSWJFFPFHRWYLYFYERILGSLIND |
| Motif 4 | Unknown | NQPLRVRPAAHLVNDEYJAKYKKALKLMKALPSDDPRNFTQQANVHCAYCDG |
| Motif 5 | PPO1_KFDV | IEFDSBDPVKFDVFINDEDDKEIGPDBTEFAGSFVTVPHS |


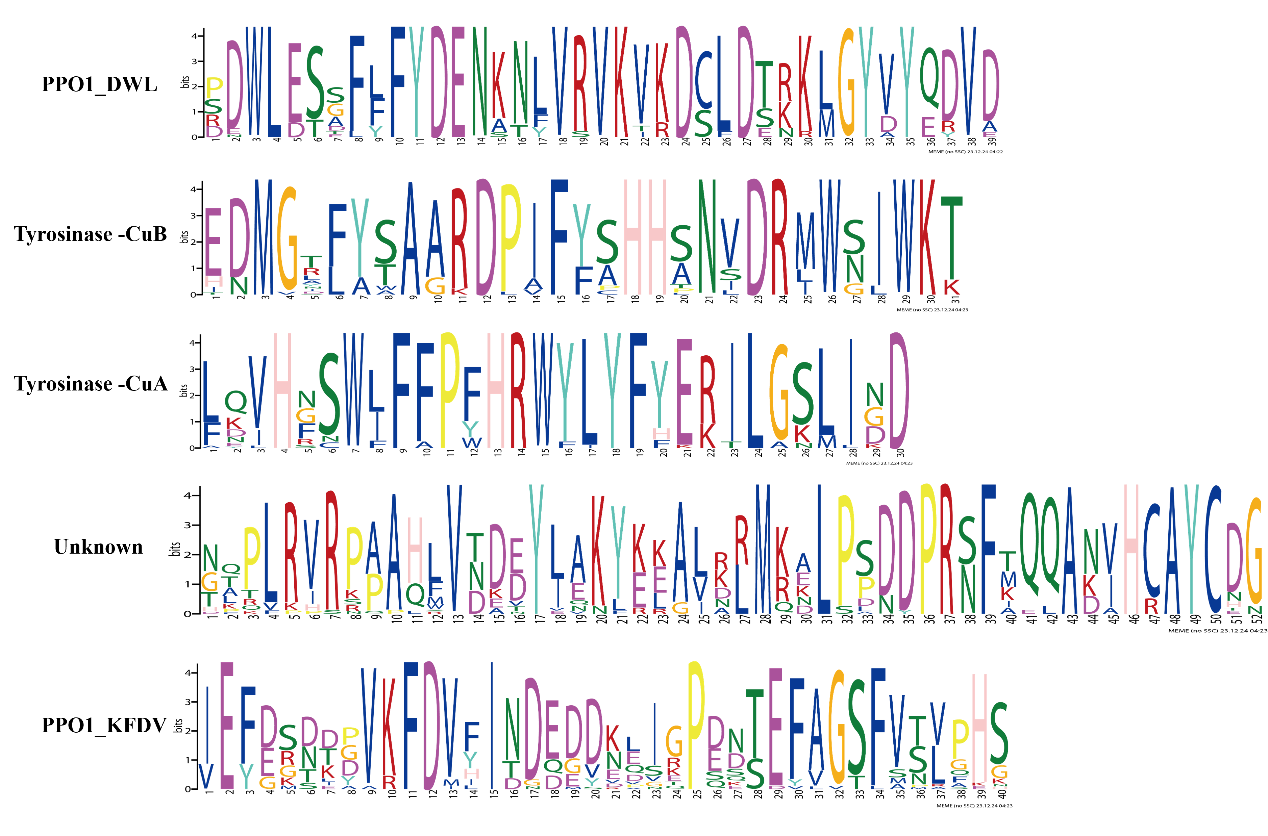


**Figure S1**. Motif visualization results

**Table S2** Intra- and inter-species covariance gene pairs in soybean and wild soybean

| **ID_1** | **ID_2** |
| --- | --- |
| *GmPPO4* | *GmPPO11* |
| *GmPPO5* | *GmPPO15* |
| *GmPPO7* | *GmPPO8* |
| *GmPPO8* | *GmPPO15* |
| *GmPPO9* | *GmPPO11* |
| *GmPPO9* | *GmPPO13* |
| *GmPPO12* | *GmPPO14* |
| *GsPPO3* | *GsPPO8* |
| *GsPPO3* | *GsPPO10* |
| *GsPPO4* | *GsPPO13* |
| *GsPPO6* | *GsPPO7* |
| *GsPPO7* | *GsPPO13* |
| *GsPPO8* | *GsPPO10* |
| *GsPPO8* | *GsPPO12* |
| *GsPPO10* | *GsPPO12* |
| *GmPPO2* | *GsPPO1* |
| *GmPPO3* | *GsPPO2* |
| *GmPPO4* | *GsPPO3* |
| *GmPPO5* | *GsPPO3* |
| *GmPPO6* | *GsPPO3* |
| *GmPPO7* | *GsPPO3* |
| *GmPPO8* | *GsPPO3* |
| *GmPPO9* | *GsPPO3* |
| *GmPPO10* | *GsPPO3* |
| *GmPPO4* | *GsPPO4* |
| *GmPPO5* | *GsPPO4* |
| *GmPPO6* | *GsPPO4* |
| *GmPPO7* | *GsPPO4* |
| *GmPPO8* | *GsPPO4* |
| *GmPPO9* | *GsPPO4* |
| *GmPPO10* | *GsPPO4* |
| *GmPPO4* | *GsPPO5* |
| *GmPPO5* | *GsPPO5* |
| *GmPPO6* | *GsPPO5* |
| *GmPPO7* | *GsPPO5* |
| *GmPPO8* | *GsPPO5* |
| *GmPPO9* | *GsPPO5* |
| *GmPPO10* | *GsPPO5* |
| *GmPPO4* | *GsPPO6* |
| *GmPPO5* | *GsPPO6* |
| *GmPPO6* | *GsPPO6* |
| *GmPPO7* | *GsPPO6* |
| *GmPPO8* | *GsPPO6* |
| *GmPPO9* | *GsPPO6* |
| *GmPPO10* | *GsPPO6* |
| *GmPPO4* | *GsPPO7* |
| *GmPPO5* | *GsPPO7* |
| *GmPPO6* | *GsPPO7* |
| *GmPPO7* | *GsPPO7* |
| *GmPPO8* | *GsPPO7* |
| *GmPPO9* | *GsPPO7* |
| *GmPPO10* | *GsPPO7* |
| *GmPPO4* | *GsPPO8* |
| *GmPPO5* | *GsPPO8* |
| *GmPPO6* | *GsPPO8* |
| *GmPPO7* | *GsPPO8* |
| *GmPPO8* | *GsPPO8* |
| *GmPPO9* | *GsPPO8* |
| *GmPPO10* | *GsPPO8* |
| *GmPPO4* | *GsPPO9* |
| *GmPPO5* | *GsPPO9* |
| *GmPPO6* | *GsPPO9* |
| *GmPPO7* | *GsPPO9* |
| *GmPPO8* | *GsPPO9* |
| *GmPPO9* | *GsPPO9* |
| *GmPPO10* | *GsPPO9* |
| *GmPPO11* | *GsPPO10* |
| *GmPPO12* | *GsPPO10* |
| *GmPPO13* | *GsPPO10* |
| *GmPPO14* | *GsPPO10* |
| *GmPPO15* | *GsPPO10* |
| *GmPPO11* | *GsPPO11* |
| *GmPPO12* | *GsPPO11* |
| *GmPPO13* | *GsPPO11* |
| *GmPPO14* | *GsPPO11* |
| *GmPPO15* | *GsPPO11* |
| *GmPPO11* | *GsPPO12* |
| *GmPPO12* | *GsPPO12* |
| *GmPPO13* | *GsPPO12* |
| *GmPPO14* | *GsPPO12* |
| *GmPPO15* | *GsPPO12* |
| *GmPPO11* | *GsPPO13* |
| *GmPPO12* | *GsPPO13* |
| *GmPPO13* | *GsPPO13* |
| *GmPPO14* | *GsPPO13* |
| *GmPPO15* | *GsPPO13* |
| *GmPPO16* | *GsPPO14* |
| *GmPPO17* | *GsPPO14* |
| *GmPPO16* | *GsPPO15* |
| *GmPPO17* | *GsPPO15* |

**Table S3** Information on cis-acting elements (Excel Table S3).

**Table S4** Phtozome database transcriptome raw data

| **GeneID** | **root** | **stem** | **leaves** | **sam** | **flower** | **pod** | **seed** | **root hairs** | **nodules** |
| --- | --- | --- | --- | --- | --- | --- | --- | --- | --- |
| *GmPPO1* | 0 | 0.000592 | 0 | 0.008327 | 0.000265 | 0.000157 | 0.001361 | 0.00014 | 0.029399 |
| *GmPPO2* | 1.7846 | 0.150357 | 8.13811 | 0.010019 | 2.74474 | 0.46071 | 0 | 0.208891 | 0.622927 |
| *GmPPO3* | 20.2612 | 4.62048 | 8.06532 | 109.901 | 8.30385 | 4.86539 | 0.185174 | 0.561778 | 0.085487 |
| *GmPPO4* | 0.944254 | 1.62261 | 0.141191 | 21.5034 | 1.35509 | 9.97308 | 0 | 0.17235 | 0.057505 |
| *GmPPO5* | 0.024615 | 0.416485 | 0 | 0.025875 | 0 | 0 | 0 | 0.945464 | 0 |
| *GmPPO6* | 1.51309 | 3.29609 | 0.004245 | 23.8776 | 16.0402 | 0.221594 | 0 | 4.64477 | 2.19286 |
| *GmPPO7* | 87.9563 | 7.27498 | 0.020348 | 113.654 | 6.34564 | 3.03095 | 0 | 31.4481 | 0.110861 |
| *GmPPO8* | 44.8454 | 21.1078 | 0.106927 | 23.2833 | 2.81419 | 0 | 0 | 43.1368 | 92.5656 |
| *GmPPO9* | 0 | 0.04828 | 45.9578 | 1.06367 | 0.100046 | 0.086672 | 0 | 0 | 0 |
| *GmPPO10* | 23.8767 | 11.1862 | 0.116459 | 101.706 | 11.2035 | 0.010677 | 0 | 22.9589 | 44.2943 |
| *GmPPO11* | 1.69139 | 0.500342 | 0.11165 | 0 | 195.026 | 0.225546 | 0 | 0.020899 | 0.158126 |
| *GmPPO12* | 0 | 0 | 0 | 0 | 0 | 0 | 0.0916 | 0 | 0 |
| *GmPPO13* | 41.5063 | 0.011329 | 0 | 0.011481 | 2.63218 | 0 | 0 | 8.55246 | 8.25324 |
| *GmPPO14* | 0 | 0 | 0 | 0 | 0 | 0 | 0 | 0 | 0 |
| *GmPPO15* | 19.8255 | 4.3167 | 21.0332 | 0.042587 | 815.675 | 0.911491 | 0 | 2.5185 | 6.6694 |
| *GmPPO16* | 2.27289 | 3.72553 | 23.1855 | 7.79511 | 0.477328 | 6.95089 | 0.016256 | 0.015832 | 0.083325 |
| *GmPPO17* | 11.9277 | 2.90173 | 0.207774 | 46.851 | 5.83173 | 3.33142 | 0.244586 | 2.32745 | 6.23566 |

**Table S5**. List of primers for qRT-PCR

| **Primers id** | **sequences** |
| --- | --- |
| GmPPO1-F | GGGGTCTATAGAGGTTGCTCC |
| GmPPO1-R | CGAGTTCAGGTGATGAGCGT |
| GmPPO2-F | ACGCTCATCACCTGAACTCG |
| GmPPO2-R | TCAATAGCTTGCTTTTCGGCG |
| GmPPO3-F | TTGGTTGGAATCTGGGCTCC |
| GmPPO3-R | TTTGAACCCTCGACCTACGC |
| GmPPO4-F | AAGCTGGTTCTGCACCTCAG |
| GmPPO4-R | CCCCATGTCGTGGTGATTGT |
| GmPPO5-F | TAGACGACCTGCACAATGGG |
| GmPPO5-R | TAGCCAATTGCCGTGGACTT |
| GmPPO6-F | ACAGCTGGACCGGTGAATTAG |
| GmPPO6-R | TGGAAGGTCAACACGTTGGT |
| GmPPO7-F | GCGGCATGGTAATCCCTCT |
| GmPPO7-R | CTACAGTCCCTCCACCGAGT |
| GmPPO8-F | AGAAAGGCTAAGAAGGTGGCG |
| GmPPO8-R | TACCAGTGTGACCAAAACATCT |
| GmPPO9-F | AGCCATAGCTGCTCCCATTC |
| GmPPO9-R | CCTAAGGGGTGCTCCAGAAG |
| GmPPO10-F | TTCTCCTTGGCATTGGAGGC |
| GmPPO10-R | GCAGGTGGTCTAACCCTGAG |
| GmPPO11-F | CAACACCAAACCCATCCGAAG |
| GmPPO11-R | GTTGACCAAATGTGCCGCTG |
| GmPPO12-F | CTGGTGCTGGCTCCTTAGAG |
| GmPPO12-R | CAGTGAAATCTCGCCCACCA |
| GmPPO13-F | CTCCAACGCTCTTCTTCGGT |
| GmPPO13-R | TCCACATTAGCGTGGTGAGA |
| GmPPO14-F | ATGACCCAACCTTCGCCATT |
| GmPPO14-R | GGGTTGATGATTGGCATCGC |
| GmPPO15-F | ATTCCCAGGGAGAACCTCCA |
| GmPPO15-R | CGGCTGGTCTTACCCTCAAG |
| GmPPO16-F | TGCCCTTAGTTTCCTTACTCAAC |
| GmPPO16-R | AAGACGTGGTTTGTCTCTTCC |
| GmPPO17-F | CATTCATTGTGTTTCTGATGCCCT |
| GmPPO17-R | GGTGGACAACAACGGGTAGAA |
| GsPPO1-F | ACGCTCATCACCTGAACTCG |
| GsPPO1-R | TCAATAGCTTGCTTTTCGGCG |
| GsPPO2-F | TTGGTTGGAATCTGGGCTCC |
| GsPPO2-R | TTTGAACCCTCGACCTACGC |
| GsPPO3-F | AAGCTGGTTCTGCACCTCAG |
| GsPPO3-R | CCCCATGTCGTGGTGATTGT |
| GsPPO4-F | TAGACGACCTGCACAATGGG |
| GsPPO4-R | TAGCCAATTGCCGTGGACTT |
| GsPPO5-F | ACAGCTGGACCGGTGAATTAG |
| GsPPO5-R | TGGAAGGTCAACACGTTGGT |
| GsPPO6-F | GCGGCATGGTAATCCCTCT |
| GsPPO6-R | CTACAGTCCCTCCACCGAGT |
| GsPPO7-F | AGAAAGGCTAAGAAGGTGGCG |
| GsPPO7-R | TACCAGTGTGACCAAAACATCT |
| GsPPO8-F | AGCCATAGCTGCTCCCATTC |
| GsPPO8-R | CCTAAGGGGTGCTCCAGAAG |
| GsPPO9-F | TTCTCCTTGGCATTGGAGGC |
| GsPPO9-R | GCAGGTGGTCTAACCCTGAG |
| GsPPO10-F | CAACACCAAACCCATCCGAAG |
| GsPPO10-R | GTTGACCAAATGTGCCGCTG |
| GsPPO11-F | CTGGTGCTGGCTCCTTAGAG |
| GsPPO11-R | CAGTGAAATCTCGCCCACCA |
| GsPPO12-F | CTCCAACGCTCTTCTTCGGT |
| GsPPO12-R | TCCACATTAGCGTGGTGAGA |
| GsPPO13-F | ATTCCCAGGGAGAACCTCCA |
| GsPPO13-R | CGGCTGGTCTTACCCTCAAG |
| GsPPO14-F | TGCCCTTAGTTTCCTTACTCAAC |
| GsPPO14-R | AAGACGTGGTTTGTCTCTTCC |
| GsPPO15-F | CATTCATTGTGTTTCTGATGCCCT |
| GsPPO15-R | GGTGGACAACAACGGGTAGAA |
| Actin1-F | CGGTGGTTCTATCTTGGCATC |
| Actin1-R | GTCTTTCGCTTCAATAACCCTA |

**Table S6** Summary of Interacting Proteins (Excel Table S6).


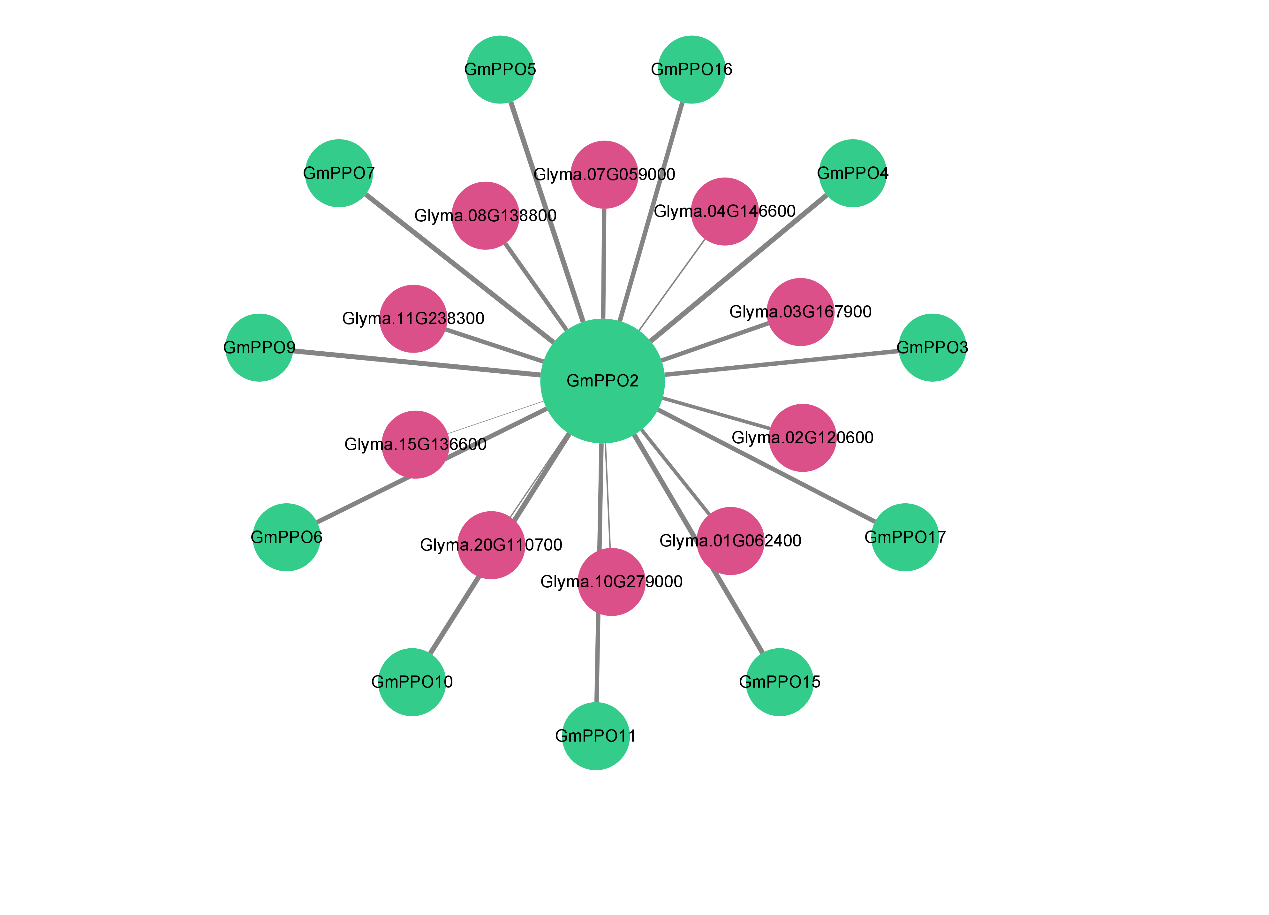


**Figure S2**. Protein–protein interaction network diagram of *GmPPO2*. The green circles represent members of the PPO family, the red circles represent interactors, and the thickness of the lines indicates the combined score.

**Table S7** Gene Nomenclature Control List

| **Our Name** | **Our ID** | **Old Name*** | **Old ID*** |
| --- | --- | --- | --- |
| GmPPO1 | Glyma.01G139800 |  |  |
| GmPPO2 | Glyma.04G121700 |  |  |
| GmPPO3 | Glyma.06G270400 | GmaPPO1 | Glyma06g42170.1 |
| GmPPO4 | Glyma.07G193300 | GmaPPO2 | Glyma07g31270.1 |
| GmPPO5 | Glyma.07G193400 | GmaPPO3 | Glyma07g31280.1 |
| GmPPO6 | Glyma.07G193500 | GmaPPO4 | Glyma07g31300.1 |
| GmPPO7 | Glyma.07G193600 | GmaPPO5 | Glyma07g31310.1 |
| GmPPO8 | Glyma.13G183000 | GmaPPO6 | Glyma13g25150.1 |
| GmPPO9 | Glyma.13G183200 | GmaPPO7 | Glyma13g25180.1 |
| GmPPO10 | Glyma.13G183500 | GmaPPO8 | Glyma13g25260.1 |
| GmPPO11 | Glyma.13G242300 | GmaPPO9 | Glyma13g31590.1 |
| GmPPO12 | Glyma.13G242400 |  |  |
| GmPPO13 | Glyma.15G071000 |  |  |
| GmPPO14 | Glyma.15G071100 |  |  |
| GmPPO15 | Glyma.15G071200 | GmaPPO10 | Glyma15g07710.1 |
| GmPPO16 | Glyma.18G225000 |  |  |
| GmPPO17 | Glyma.18G225100 | GmaPPO11 | Glyma18g45900.1 |

* Tran, L.T.; Taylor, J.S.; Constabel, C. The polyphenol oxidase gene family in land plants: Lineage-specific duplication and expansion. BMC Genomics 2012, 13, 395.
